# Clinical characteristics and outcomes of patients with Herpes Simplex Encephalitis in Vietnam: a retrospective study

**DOI:** 10.1186/s12879-024-09453-3

**Published:** 2024-06-03

**Authors:** Ta Thi Dieu Ngan, Nguyen Thi Tuyet, Dinh Trong Hung, Nguyen Trung Cap, Duy Manh Nguyen, Vu Quoc Dat

**Affiliations:** 1 Department of Infectious Diseases, Hanoi Medical University, 1 Ton That Tung Street, Dong Da district, Hanoi, Vietnam; 2 National Hospital for Tropical Diseases, 78 Giai Phong Street, Dong Da District, Hanoi, Vietnam; 3 Hanoi Medical University Hospital, 1 Ton That Tung Street, Dong Da District, Hanoi, Vietnam; 4 Thai Nguyen University of Medicine and Pharmacy, 284 Luong Ngoc Quyen Street, Thai Nguyen City, Thai Nguyen Province Vietnam; 5 Grinnell College, 1115 8th Avenue, Grinnell, IA 50112 USA

**Keywords:** Herpes simplex encephalitis, Prognostic factors, Outcome, Severity

## Abstract

**Background:**

Herpes simplex encephalitis (HSE) is an important central nervous infection with severe neurological sequelae. The aim of this study was to describe clinical characteristic and outcomes of patients with HSE in Vietnam.

**Methods:**

This was a retrospective study of 66 patients with herpes simplex encephalitis who admitted to the National Hospital for Tropical Diseases, Hanoi, Vietnam from 2018 to 2021. The detection of herpes simplex virus (HSV) in cerebrospinal fluid was made by the real-time PCR assay. We reported the clinical manifestation on admission and evaluated clinical outcomes at the hospital discharge by modified Rankin Scale (mRS). Multivariate logistic regression analysis was used to analyze the independent risk factors of severe outcomes.

**Results:**

Of the 66 patients with laboratory confirmed HSE, the median age was 53 years (IQR 38–60) and 44 patients (69.7%) were male. The most common manifestations included fever (100%), followed by the consciousness disorder (95.5%). Other neurological manifestation were seizures (36.4%), memory disorders (31.8%), language disorders (19.7%) and behavioral disorders (13.6%). Conventional magnetic resonance imaging (MRI) showed 93.8% patients with temporal lobe lesions, followed by abnormalities in insula (50%), frontal lobe (34.4%) and 48.4% of patients had bilateral lesions. At discharge, 19 patients (28.8%) completely recovered, 15 patients (22.7%) had mild sequelae, 28 patients (42.4%) had moderate to severe sequelae. Severe neurological sequelae were memory disorders (55.8%), movement disorders (53.5%), language disorders (30.2%). Multivariate logistic regression analysis showed that Glasgow score decrement at admission, seizures, and time duration from onset of symptoms to the start of Acyclovir treatment > 4 days were independent factors associated with severe outcomes in HSE patients.

**Conclusion:**

Glasgow score decrement, seizures and delay treatment with Acyclovir were associated with the poor outcome of patients with HSE.

## Introduction

Herpes simplex virus (HSV) encephalitis is a life-threatening central nervous system infection associated with poor outcomes [1]. It occurs sporadically with a frequency of 2–4 cases per 1,000,000 population per year in high-income countries [2]. Over 90% of HSE cases are caused by HSV-1, with the remainder attributed to HSV-2 [3].

Prior to the availability of effective antiviral treatment, the mortality rate of encephalitis caused by HSV was about 70% [4]. In the 1980s, the introduction of intravenous Acyclovir significantly improved the prognosis of HSE, reducing the mortality rate to only 6-11% [3]. In the 1990s, the use of polymerase chain reaction (PCR) for HSV detection in the cerebrospinal fluid further enhanced early prognosis and treatment [5]. However, the rate of neurological sequelae remains high, happening in 44–62% of the surviving patients [6] with the delay in antiviral initiation as the most common reason [7–9].

In the context of Vietnam, HSE constitutes 7% of pediatric cases with acute encephalitis [10] and around 3% of suspected central nervous system (CNS) infections in adults [11]. However, research on this severe condition, both domestically and internationally, is restricted, due to the low incidence of this disease and the prevalence of small-scale studies primarily focusing on clinical symptoms. This study aims to address this gap by providing a comprehensive analysis of the clinical characteristics and prognostic factors linked to poor outcomes in HSE patients using a substantial cohort of cases from 2018 to 2021 at the National Hospital for Tropical Diseases (NHTD), a tertiary hospital specialized in treating infectious diseases.

## Materials & methods

### Study design

This retrospective study analyses medical records of all patients who were diagnosed with HSE at the National Hospital for Tropical Diseases (NHTD), which is a referral hospital for treatment of infectious diseases in the North of Vietnam, from July 1, 2018, to June 30, 2021. The inclusion criteria of patients included: (1) over 18 years of age, (2) suspected encephalitis according to the guidelines of the International Encephalitis Association (2013) [12] and (3) had positive HSV DNA in cerebrospinal fluid tests using Real-time PCR assay (Bio-Rad *Iq5 Multicolor Real Time PCR* Detection *System* and *Chromo4 real-time PCR* detection system). Criteria for suspected encephalitis consisted of major criterion of altered mental status (defined as decreased or altered level of consciousness, coma, or personality changes) lasting ≥ 24 h with no other identifiable cause and at least of 2 minor criteria of fever, generalized or partial convulsions, new onset of focal neurologic findings, CSF with lymphocytosis (≥ 5 white blood cells/µl) and brain parenchymal abnormalities on neuroimaging or EEG suggestive of encephalitis [12]. Patients with pre-existing brain injury, history of mental disorders, inability to communicate normally (mute or deaf), or concurrent bacterial meningitis were excluded from the study. The protocol of this study was approved by the Ethics Committee and review board of the National Hospital for Tropical Diseases (IORG0006480) with approval number 9 A/HDDD-NĐTƯ dated 22, June 2021. As the study was conducted retrospectively, the informed consent waiver was accepted by the Ethics Committee and review board of the National Hospital for Tropical Diseases.

### Data collection and outcome assessment

We collected information of (1) demographics, (2) clinical symptoms and signs, (3) results of laboratory testing and cerebrospinal fluid analysis at admission and during the hospitalization, (4) reports of brain MRI and CT, and (5) treatment (Acyclovir, mechanical ventilator).

The patient’s outcomes at the time of discharge were evaluated using the modified Rankin scale (mRS) [13]. The scores of mRS were classified as follow: 0 (No sequelae), 1 (No significant sequelae despite having symptoms, capable of performing all usual tasks and activities), 2 (Slight sequelae, unable carry out all previous activities, but able to look after own affairs without assistance), 3 (Moderate sequelae, requiring some help but able to walk without assistance), 4 (Moderately severe sequelae, unable to wall or attend to own bodily needs without assistance), 5 (Severe sequelae, being bedridden, incontinent and requiring constant nursing care or attention) and 6 (Dead). The outcomes of the patients were categorized as mild if the mRs score was < 3 or as severe if the mRS score was ≥ 3.

### Statistical analysis

SPSS software version 20.0 was used for statistical analysis. Descriptive analysis was performed to explore the demography, clinical manifestations, and laboratory results. As the data were not normally distributed, non-parametric tests were used for statistical analysis, specifically the Mann-Whitney U test to compare the median of the quantitative variables and χ2 test to compare the proportions of the qualitative variables. While a preliminary analysis was published on a subset of participants in Vietnam Medical Journal [14] this work was performed on the completed sample using the univariate and multivariate logistic regression analysis to explore the independent factors associated with disease severity. The statistically significant cutoff was *p* < 0.05.

## Results

### Patient characteristics

66 eligible patients were included in the study, with a median age of 53 (IQR, 38–60 years), of whom 46 patients (69.7%) were male. There were 30.3% of patients who had at least one comorbidity, in which the most common comorbidity was cardiovascular diseases (18.2%), followed by diabetes (9.1%). Most of the patients (90.9%) were transferred from primary and secondary-level hospitals to the NHTD. The median time from the onset of symptoms to hospital admission was approximately 5.5 days (IQR 3–7 days) while the median time from the onset of symptoms to administration of Acyclovir was 6.0 days. The clinical manifestation and laboratory results were showed in the Table 1. Fever was presented in all patients while meningeal signs were apparent in 57 patients (86.4%). 63 patients (95.5%) were admitted with disturbed consciousness, in which 24 patients (36.4%) had seizures. The median of the GCS is 13, which has been rounded up to preserve its clinical significance. 25 patients (37.9%) required admission to the intensive care unit, in which the majority of patients required mechanical ventilation and developed ventilator associated pneumonia.

**Table 1:** Clinical manifestations and laboratory tests on admission

Characteristics		Value
**Clinical and blood tests**		
Fever		66 (100%)
Seizures (n, %)		24 (36.4%)
Memory disorders (n, %)		21 (31.8%)
Language disorders (n, %)		13 (19.7%)
Behavioral disorders (n, %)		9 (13.6%)
Consciousness disorders (n, %)		63 (95.5%)
GCS (median, IQR)		13 (12–14)
Meningeal signs (n, %)		57 (86.4%)
Signs of hemiplegia (n, %)		5 (7.6%)
WBC > 10G/l (n, %)		42 (63.6%)
Blood sodium < 130 mmol/l (n, %)		25 (37.9%)
CRP > 20 mg/l (*n* = 63)		16 (25.4%)
Abnormal MRI findings (*n* = 64)		61 (95.3%)
Bilateral lesion on MRI		31 (48,4%)
**Cerebrospinal fluid test**		
Cells (cells/mm3)	Cells ≤ 5	1 (1.5%)
	5 < cells ≤ 200	41 (62.1%)
	200 < cells ≤ 500	17 (25.8%)
	Cells > 500	7 (10.6%)
Protein (g/L)	≤ 0,45	14 (21.2%)
	0,45–1	33 (50%)
	> 1	19 (28.8%)

GCS = Glasgow Coma Score | WBC = White Blood Cells | MRI = Magnetic Resonance Imaging | CRP = C-reactive protein

64 patients (97%) had brain MRI results. The median duration from the onset of symptoms to the MRI examination was 6 (IQR, 4–9) days with the common lesions being observed in the temporal lobe (93.8%), insular lobe (50%), and frontal lobe (34.4%).

**Fig. 1:**
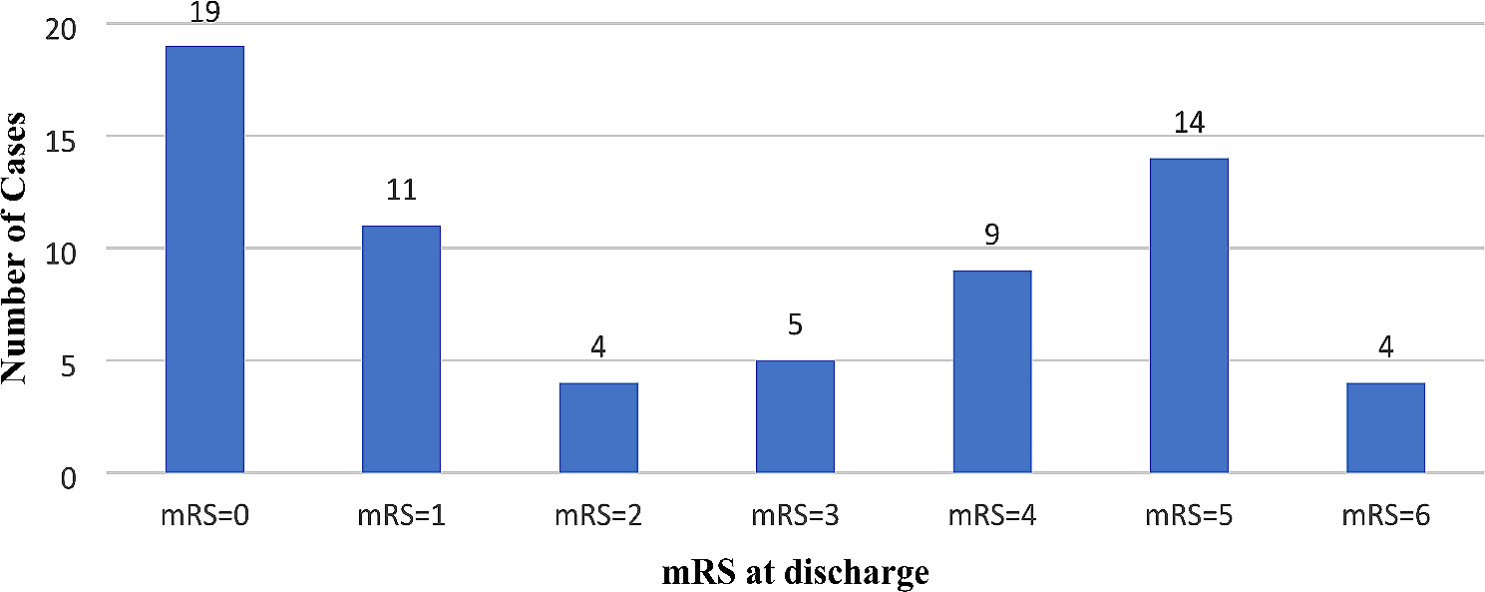
Outcomes at discharge according to the mRS

Figure 1. illustrates patient outcomes at discharge: 28.8% achieved full recovery (mRS = 0), 22.7% had mild sequelae (0 < mRS ≤ 2), 42.4% experienced moderate to severe sequelae (3 ≤ mRS ≤ 5), and 4 deaths. Of the 43 patients with sequelae, common symptoms included memory disorders (55.8%), movement disorders (53.5%), and language disorders (30.2%).

### Prognostic factors for severe outcome

The differences in the frequency of symptoms and laboratory results on admission according to mRS were showed in the Tables 2 and 3. The patients with mRS score ≥ 3 had higher rate of seizures (56.2% vs. 17.6%), lower GCS score (12 points vs. 13 points), higher rate of hemiplegia (15.6% vs. 0%), and more frequency of multiple lobar involvements in MRI (46.7% vs. 14.7%).

**Table 2:** Comparison of clinical manifestations at admission by the mRS groups

	Mild cases, mRS < 3 (*n* = 34)	Severe cases, mRS *≥* 3 (*n* = 32)	*P*
Headache (n, %)	33 (97.1%)	31 (96.9%)	1.000
Nausea, vomiting (n, %)	22 (64.7%)	16 (50%)	0.227
Seizures (n, %)	6 (17.6%)	18 (56.2%)	**0.001**
GCS (median, IQR)	13 (13–14)	12 (10–13)	**0.003**
Memory disorders (n, %)	13 (38.2%)	8 (25%)	0.249
Language disorders (n, %)	8 (23.5%)	5 (15.6%)	0.420
Behavioral disorders (n, %)	3 (8.8%)	6 (18.8%)	0.297
Meningeal signs (n, %)	30 (88.2%)	27 (84.4%)	0.066
Signs of hemiplegia (n, %)	0 (0%)	5 (15.6%)	**0.023**
White blood cells (G/l) (Median, IQR)	10.25 (6.3–11.2)	11.4 (10.2–13.0)	**0.001**
Platelets (G/l) (Median, IQR)	174.5 (152.3–217)	179 (156.5-230.8)	0.768
AST (UI/L) (Median, IQR)	28.2 (22.9–39.5)	41 (30.3–71.3)	**0.026**
ALT (UI/L) (Median, IQR)	20.9 (13.2–36)	38.5 (22.5–53.3)	**0.008**
CSF cells (cells/mm3) (Median, IQR)	159 (90–370)	136 (40–297)	0.253
CSF fluid protein (g/l) (Median, IQR)	0.7 (0.5–1.1)	0.7 (0.5–1.2)	0.827
CSF glucose (mmol/l) (Median, IQR)	3.9 (3.4–4.9)	4.3 (3.7–4.7)	0.464
CSF chloride (mmol/l) (Median, IQR)	117.5 (113.8–119)	117. 5 (113.3–121)	0.268

GCS = Glasgow Coma Score | ALT = Alanine aminotransferase | AST = Aspartate aminotransferase | CSF = Cerebrospinal fluid

**Table 3:** Comparison of cranial MRI results according to the mRS

		Mild cases (*n* = 34) (*n*, %)	Severe cases (*n* = 30) (*n*, %)	*P*
Location of abnormalities	Temporal lobes	32 (94.1%)	28 (93.3%)	1.000
	Insula	14 (41.2%)	18 (60%)	0.133
	Frontal lobe	7 (20.6%)	15 (50%)	**0.013**
	Parietal lobes	2 (5.9%)	6 (20%)	0.133
	Occipital lobe	2 (5.9%)	2 (6.7%)	1.000
Lesions ≥ 3 lobes		5 (14.7%)	14 (46.7%)	**0.005**
Cerebral edema		2 (5.9%)	6 (20%)	0.133

Multivariate logistic regression analysis showed that lower GCS at admission (aOR: 0.25–0.76 per 1 point increment), seizure (aOR 1.65–37.14) and time from onset to the start of Acyclovir treatment > 4 days (aOR 1.48–44.31) were independent factors related to severe prognosis in patients with HSE, as shown in Table 4.

**Table 4:** Univariable and multivariable analysis for severe prognostic factors

	Univariate analysis			Multivariate analysis		
	OR	95%CI	*P*	OR	95%CI	*P*
Age > 40	0.91	0.32–2.62	0.871	-	-	
Male	2.21	0.75–6.55	0.152	-	-	
GCS at admission (+ 1 point)	0.55	0.36–0.82	**0.004**	0.44	0.25–0.76	**0.004**
Seizure	6.0	1.95–18.48	**0.002**	7.84	1.65–37.14	**0.01**
Frontal lobe lesion on MRI	3.86	1.29–11.55	**0.016**	1.44	0.19–11.08	0.727
Lesions ≥ 3 lobes on MRI	5.08	1.54–16.68	**0.007**	4.61	0.53–40.44	0.168
Time between onset of symptoms and Acyclovir being administered > 4 days	3.18	1.08–9.30	**0.035**	8.10	1.48–44.31	**0.016**

GCS = Glasgow Coma Score | MRI = Magnetic Resonance Imaging | CRP = C- reactive protein

## Discussion

A study between 2014 and 2017 in Vietnam reveals that the main pathogens responsible for CNS infections among 137 patients were *Streptococccus suis* (12%) and *Neisseria meningitidis* (7%), followed by HSV (3%) [15]. Similarly, another study in Southern Vietnam over the duration of 12 years (1996–2008) found that HSV ranked 2nd among viral etiologies for CNS infection (6.5%) after Japanese encephalitis virus (12%) [16]. Our study is the first to focus on HSE in the Northern Vietnamese regions with a significant number of patients. Within the scope of our research, we found that the in-hospital mortality was at 6.1%, which was similar to the those reported by studies in both France (5.5%) [17] and India (6.8%) [18]. Additionally, we also identified an association between poor outcomes in HSE patients and neurological symptoms as well as delays in antiviral initiations, serving as potential prognosis factors.

Among the symptoms, consciousness status at the time of admission determined by the GCS score is strongly associated with poor outcomes in HSE patients. According to our analysis, the lower the GCS, the higher the risk of being discharged from hospital in severe condition. The probability of hospital discharge with severe conditions decreases by 0.44 times when GCS increases by 1 point. This result is in accordance with that of the study by Kamei et al., in which the same probability decreases by 1.45 times per 1 increment of GCS score before treatment [19]. Although we did not focus on the threshold of GCS as a prognostic factor, previous reports have discussed this. A multinational study by Erdem et al. in 2015 with 438 patients showed that those with a GCS score of less than 5 experienced poor outcomes more frequently [20]. Similarly, Whitley also assessed the prognostic capability of GCS score across all age ranges and confirmed that a GCS score of less than 6 can predict a poor outcome in HSE patients, irrespective of the medicine administered or the age of the patients [21].

Along with worsening consciousness, seizure is another clinical manifestation of HSE patients, with an incidence rate ranging from 35 to 65% [22]. Our analysis indicates that seizure on admission is an independent prognosis of severity. In particular, patients with seizures are 7.84 times more likely in a severe condition than those without. Therefore, the presence of seizures should also be closely monitored in HSE patients.

In treating HSE patients, the administration of Acyclovir has been reported to be of paramount importance, especially during the early stage of the disease [8, 20, 23, 24]. Correspondingly, our analysis found that patients who received Acyclovir 4 days after the onset of symptoms are 8.1 times more likely to be in severe conditions, indicating that it is more beneficial for patients to be administered Acyclovir early. However, the benefits of the empirical use of Acyclovir in patients likely diagnosed with HSE without any confirmation from molecular tests such as HSV CFS PCR tests have not been clinically proven. In the study by Benson and Swadron, 17 out of 24 patients (71%) with suspected HSE was not administered with Acyclovir in the emergency department but rather in in-patient settings, though the morbidity and mortality of the patients were not examined due to the short study length [25]. In addition, despite having high sensitivity and specificity [21], standard HSV CFS PCR test requires long time for confirmation and also poses possibility of false positives [26, 27], leading to our proposal that the decision to treat HSE early should be based on clinical evaluation and brain imaging rather than deferring treatments until the PCR result is available.

Due to the retrospective nature of our study, we were restricted to evaluate the outcomes of the patients at the time of hospital discharge, and we could not follow up on the sequelae caused by HSE for a longer term. It is also probable that there are some restrictions in comparing the clinical characteristics because the results did not correspond to the disease progression of the patients included in the study as well as the referral system from tertiary healthcare centers might cause delays in recording such characteristics.

## Conclusion

HSE was associated with poor outcome and high case-fatality at hospital discharge in Vietnamese patients. This study highlights the importance of early diagnosis and empirical treatment in the lower level of healthcare system in Vietnam to improve the clinical outcome.

## Data Availability

The dataset used and/or analyzed during the current study are available from the corresponding author on reasonable request.

## Abbreviations

ALT: Alanine aminotransferase
AST: Aspartate aminotransferase
CNS: Central nervous system
CSF: Cerebrospinal fluid
CT: Computed tomography
CRP: C reactive protein
HSE: Herpes simplex encephalitis
HSV: Herpes simplex virus
GCS: Glasgow Coma Score
MRI: Magnetic resonance imaging
mRS: Modified Rankin Scale
NHTD: National Hospital for Tropical Diseases
PCR: Polymerase Chain Reaction
WBC: White blood cell
